# The Usability of Sorbents in Restoring Enzymatic Activity in Soils Polluted with Petroleum-Derived Products

**DOI:** 10.3390/ma16103738

**Published:** 2023-05-15

**Authors:** Jadwiga Wyszkowska, Agata Borowik, Magdalena Zaborowska, Jan Kucharski

**Affiliations:** Department of Soil Science and Microbiology, Faculty of Agriculture and Forestry, University of Warmia and Mazury in Olsztyn, Plac Łódzki 3, 10-727 Olsztyn, Poland; agata.borowik@uwm.edu.pl (A.B.); m.zaborowska@uwm.edu.pl (M.Z.); jan.kucharski@uwm.edu.pl (J.K.)

**Keywords:** organic pollutants, soil quality, plant biomass, molecular sieve, expanded clay, sepiolite, Ikasorb

## Abstract

Due to their ability to adsorb or absorb chemical pollutants, including organic compounds, sorbents are increasingly used in the reclamation of soils subjected to their pressure, which results from their high potential in eliminating xenobiotics. The precise optimization of the reclamation process is required, focused primarily on restoring the condition of the soil. This research are essential for seeking materials sufficiently potent to accelerate the remediation process and for expanding knowledge related to biochemical transformations that lead to the neutralization of these pollutants. The goal of this study was to determine and compare the sensitivity of soil enzymes to petroleum-derived products in soil sown with *Zea mays*, remediated using four sorbents. The study was conducted in a pot experiment, with loamy sand (LS) and sandy loam (SL) polluted with VERVA diesel oil (DO) and VERVA 98 petrol (P). Soil samples were collected from arable lands, and the effects of the tested pollutants were compared with those used as control uncontaminated soil samples in terms of *Zea mays* biomass and the activity of seven enzymes in the soil. The following sorbents were applied to mitigate DO and P effects on the test plants and enzymatic activity: molecular sieve (M), expanded clay (E), sepiolite (S), and Ikasorb (I). Both DO and P exerted a toxic effect on *Zea mays*, with DO more strongly disturbing its growth and development and the activities of soil enzymes than P. In sandy clay (SL), P was found to be a significant inhibitor of dehydrogenases (Deh), catalase (Cat), urease (Ure), alkaline phosphatase (Pal), and arylsulfatase (Aryl) activities, while DO stimulated the activity of all enzymes in this soil. The study results suggest that the sorbents tested, mainlya molecular sieve, may be useful in remediating DO-polluted soils, especially when alleviating the effects of these pollutants in soils of lower agronomic value.

## 1. Introduction

Environmental pollution poses the most severe threat to the health and welfare of the global population [1,2]. This threat stems from progressing industrialization and urbanization, both being sources of pollutant emissions, which in turn exert an adverse impact on soil properties, various ecosystems as well as health of soils and human populations [3,4]. Within the last two decades, the worldwide death rate due to environmental pollution has increased by 66%. According to the Lancet Commission on Pollution and Health, pollution is the cause of approximately nine million deaths annually, which accounts for one sixth of the total deaths recorded worldwide [4]. One of the most frequent environmental challenges stems from the pollution with petroleum-derived products [5,6,7]. Annually, 1.7–8.8 million metric tons (MMT) of petroleum compounds are discharged into the environment. A total of 90% of this is related to human activity [8]. In addition, according to the IEA Oil Market Report [9], global demand for crude oil will increase to a record level of 101.9 mb d^−1^. Based on estimates published by the Organization of the Petroleum Exporting Countries [10], global oil supplies in 2021 will increase to 95.06 mb d^−1^, and production to 26.32 mb d^−1^. Alkanes, cycloalkanes and polycyclic aromatic hydrocarbons classified as petroleum hydrocarbons are considered to be the main environmental pollutants due to their stability and durability in soil ecosystems [11,12].

Estimates shows 2.8 million sites across Europe to be potentially polluted with these products [13,14], mainly due to exploration, especially in oil-producing regions; leaks from ruptured pipelines, underground and above-ground fuel tanks; handling, transport and improper discharge of hydrocarbon wastewater [15,16]. Vehicle exhaust, waste engine oil, oil spills and petroleum sludge are important sources of soil contamination with petroleum products [8]. An important problem is the complex composition of petroleum compounds related to alkanes, aromatic hydrocarbons, resins and asphaltenes. Their toxicity to organisms generates environmental transport, chemical properties and polluting nature [17].

Therefore, the penetration of petroleum-derived products into soil significantly impairs the functions of ecosystems by disturbing their microbiological [18,19,20], chemical [21] and physical properties [22]. These pollutants modify not only the composition of soil microbial communities [23,24] but may also lead to permanent soil damage, soil texture, structure and decreased pore spaces; saturated hydraulic conductivity by contributing to the loss of its fertility and productivity [22,25,26,27,28]; and diminished plant biomass production [21,29,30,31]. These adverse changes raise serious concerns due to the fact that soil is the core of human and animal nutrition [32]. Its pollution not only results in the reduced global production of food but also makes it less suitable for consumption because of deteriorated quality [29,33,34], which is deemed to be especially important in the context of dynamic demographic transformations [35]. Landrigan et al. [36] have estimated 500,000 premature death cases recorded worldwide annually to be due to the effect of soil pollution. According to the International Energy Agency (IEA), benzene (C_6_H_6_), toluene (C_7_H_8_), ethylbenzene (C_6_H_5_CH_2_CH_3_) and xylene (CH_3_)_2_C_6_H_4_ as chemical compounds adversely affect the health of humans, animals and plants. They are carcinogenic, and their composition includes more than a thousand organic substances of various toxicity. Some hydrocarbons are able to bioaccumulate, and then, as a result of adsorption and concentration, they can accumulate in topic chains and enter the human body [37,38]. Across the entire food chain, children and the elderly are the most vulnerable to the effects of soil toxins.

Hence, a critical element of managing the risk related to environmental pollution, including soil pollution, is to adapt measures provisioned in the EU strategy, driven by the “zero pollution” theory. It assumes that by 2050, soil pollution should be so low that it no longer poses threat to human health [39]. The choice of an effective method for the remediation of soils polluted with petroleum-based products seems essential in this context. It is, however, a complex procedure, because these products are blends of many pollutants, dominated by hydrocarbons [40,41]. In recent years, growing attention has been paid to innovative technologies enabling the remediation of environments contaminated with petroleum products [8,42,43]. Currently, among the methods of the reclamation of soils contaminated by petroleum-derived products, mainly physical, chemical and biological methods are proposed [44]. Physical remediation requires the implementation of countermeasures, such as vapor extraction, flotation, ultrasound, electrokinetic remediation, thermal desorption and carbon adsorption [45]. However, most of these methods have their limitations. The flotation process generates large amounts of wastewater, and its productivity decreases in weathered soil [46]. The use of ultrasound is expensive due to the energy consumption for acoustic generation [47]. In turn, the use of the electrokinetic method is unfounded at low concentrations of soil contamination [48]. Chemical methods include plasma oxidation [49] and photocatalytic degradation. However, the course of photocatalytic degradation is significantly influenced by the light absorption properties and soil moisture [50]. Bioremediation based on the action of biological factors, including microorganisms, plants or plant remains [48,50] guarantees minimal soil disturbance, no secondary pollution and reduction of greenhouse gases [51,52]. However, salinity, oxygen availability and changing pH values can inhibit the growth of microorganisms and metabolism, and thus reduce the effectiveness of bioremediation [53]. Ossai et al. [54] have emphasized that the choice of the recultivation method should be driven not only by its production effectiveness but also by its economic viability. In addition, the proposed remediation method should be scientifically justified, sustainable, non-invasive and environmentally friendly. The above criteria are met by in situ remediation techniques that use sorbents to bind heavy metals in the soil [55,56,57,58]. The unique properties of sorbents, such as the microporous structure, large specific surface area, hydrophobicity, high anion and cation adsorption capacity, as well as high thermal and chemical stability [59,60], make them suitable in in situ technologies for the reclamation of an environment contaminated with petroleum products. Silanol groups (Si-OH) located on the surface of the mineral are responsible for the effectiveness of the molecular sieve [61]. The mechanism of the absorption process is based on electrostatic interactions and ion exchange on the Si surface [62]. The mesoporous pore provides enough space for the chemical modification of the grafted functional groups, and the appropriate wall thickness causes it to exhibit high mechanical and hydrothermal stability [63]. Sepiolite is formed as a result of the diagenetic and hydrothermal alteration of rocks. Its foundation is formed from [SiO_4_]^4−^ tetrahedrons, connected at three corners by shared oxygen anions, thus forming the tetrahedral sheet. Due to its natural morphology, sepiolite is considered an agent with outstanding sorption abilities [64]. The potential of expanded clay as a bioremediation agent is based on the double porosity of this mineral, both in the intercrystallite space and in the porous core [65]. Ikasorb is a porous material derived from roasted diatomaceous earth. The sorption properties of silica adsorbents result from their high porosity and the presence of surface hydroxyl groups [66].

In the light of the above information, the monitoring and assessment of risk factors related to soil pollution is undoubtedly justified [25]. Indicators that respond very quickly to environmental stress are very important in assessing the risk of the biotic environment [67,68]. Soil enzymes, which are ubiquitous in the soil and immediately respond to environmental destruction, are very good indicators of the stability and quality of soil ecosystems [69,70]. They catalyze biochemical processes in the soil [71], promote the mineralization and decomposition of soil organic matter as well as the release of inorganic nutrients, meeting the nutritional demands of plants [72,73]. They are also capable of transforming toxic chemical substances [74,75]. Most often analyzed soil enzymes include oxidoreductases and hydrolases [68,76]. According to Dindar et al. [75] and Keller et al. [77], the determinations of the activities of soil enzymes involved in C, N and P conversion may provide some insight into metabolic capabilities of the soil, based on which it is possible to assess the potential for transformation of specified sources of energy or nutrients. Therefore, today, analyses of enzymatic activity in extreme environments, including salinated environments [78] and those polluted with heavy metals [69], phenols [79,80], plant protection agents [81,82] and petroleum-derived products [75,83,84], have attracted global interest.

It is of great importance for the protection of soil resources, environmental management and sustainable development to understand the biochemical transformations affecting the quality of soil in an ecosystem polluted with these products [75]. Considering the increasingly appreciated potential of sorbents in restoring the balance of soil contaminated with xenobiotics against the background of many chemical and physical methods, the aim of the research was established. In a conducted experiment, the responses of seven soil enzymes, including dehydrogenases, catalase, urease, acid phosphatase, alkaline phosphatase, arylsulfatase, *β*-glucosidase and *Zea mays*, to pressure caused by diesel oil and petroleum in soil submitted for remediation through the application of a molecular sieve, expanded clay, sepiolite and Ikasorb.

## 2. Materials and Methods

### 2.1. Study Design

The study was conducted in a greenhouse of the Warmia and Mazury University in Olsztyn (NE Poland, 53.72° N, 20.42° E) in the form of a pot experiment. The subject of the study included brown eutrophic soils (Eutric Cambisols) with the particle composition of loamy sand (LS) and sandy loam (SL) [85]. Their characteristics are given in Table 1. The soils derived from the area of the Olsztyn Lake District belonging to the East European Plain, which is devoid of the pressure of anthropogenic factors, and their samples were collected from the accumulation stratum (Ap) from winter rape crops. The full characteristics of the molecular sieve, expanded clay and sepiolite have been presented in our earlier publications [56,57], as were those of the sorbent Ikasorb [86].

*Zea mays* was used as the test plant. Its choice was driven by its high adaptability to stressful environmental conditions, short life cycle, ease of cultivation, broad distribution across the globe and high biomass productivity [87,88]. Morales-Máximo et al. [89] and Liao et al. [90] emphasized that maize biomass produced on polluted areas can be used in alternative energy production. Analyses were carried out in 5 replications. The aerial parts and roots of *Zea mays* were harvested at the BBCH 59 stage. Immediately after plant harvest, soil samples were collected for biochemical analyses. The samples were sifted through a screen with mesh diameter of 2 mm.

### 2.2. Methodology of Physicochemical and Chemical Determinations

In the collected soil samples, the following were determined: fraction size by aerometric method; pH in 1 mol KCl dm^-3^; hydrolytic acidity (HAC); and the sum of base exchangeable cations (EBCs).The HAC and EBC values obtained were used to compute the cation exchange capacity (CEC) and alkaline cation saturation (ACS). In addition, analyses were conducted to determine contents of total nitrogen (N_tot_), organic carbon (C_org_) and available P, K and Mg. The above determinations were conducted in 3 replications following the methods described in our previous work [79].

### 2.3. Methodology of Determination of the Activity of Soil Enzymes

The soil samples with natural moisture content were determined for the activities of the following enzymes: dehydrogenases (Deh), catalase (Cat), urease (Ure), acidic phosphatase (Pac), alkaline phosphatase (Pal), *β*-glucosidase (Glu) and arylsulfatase (Aryl). The activity of each of the individual enzymes was determined in a weighed soil sample, incubated for a designated period of time and at a constant temperature. The activity of each of the individual enzymes was determined in a weighed soil sample, incubated for a designated period of time and at a constant temperature. The parameters of each sample were as follows: for Deh—6 g of soil, 24 h of incubation (37 °C); for Cat—2 g of soil, 20 min of incubation (21 °C); for Pac, Pal, Glu and Aryl—1 g of soil, 1 h of incubation (37 °C); and for Ure—10 g of soil, 24 h of incubation (37 °C).

All determinations were made in triplicate. The following chemical compounds were used as substrates: 2,3,5-triphenyltetrazolium chloride for dehydrogenases; hydrogen peroxide for catalase; urea for urease; 4-nitrophenyl phosphate disodium salt 6-hydrate for phosphatases; potassium 4-nitrophenyl sulfate for arylsulfatase; and 4-nitrophenyl-*β*-D-glucopyranoside for *β*-glucosidase. Enzyme activity units were expressed in moles or in the subunits of their reaction products produced within 1 h in 1 kg of soil, i.e., formazane in the case of dehydrogenases, O_2_ in the case of catalase, N-NH_4_ in the case of urease, as well as 4-nitrophenol in the case of acidic and alkaline phosphatase, arylsulfatase and b-glucosidase. The extinction of the reaction products was measured using a Perkin-Elmer Lambda 25 spectrophotometer (Peabody, MA, USA) at the following wavelengths: 485 nm (dehydrogenases), 410 nm (urease, acidic and alkaline phosphatase), 420 nm (arylsulfatase) and 400 nm (*β*-glucosidase). Detailed procedures of enzyme determinations are provided in our previous works [56,91]. 

### 2.4. Calculations and Statistical Analysis

In order to determine the impact of soil pollution with petroleum-derived products and sorbents (Ad) on the biomass of aerial parts and roots of *Zea mays* and on the activities of individual soil enzymes, the index of influence (IF) of DO, P and Ad on these dependent variables was determined in this study. The IF was computed from Formula (1).

(1) $$\mathrm{IFy}=\frac{\mathrm{Po}}{\mathrm{Co}}-1$$

where

y—respectively: DO, P or sorbent (Ad);
Po—weight of *Zea mays* biomass or activity of the analyzed enzyme in the soil polluted with DO or P;
Co—weight of *Zea mays* biomass or activity of the analyzed enzyme in the control soil (uncontaminated).

The index value of 1 denotes a 100% increase in the value of the analyzed trait, whereas the index value of −1 indicates a 100% decrease in the value of the analyzed trait.

Values of the computed indices were presented on heat maps, developed using R v1.2.5033 software (Boston, MA, USA) [92] with R v3.6.2 addition [93] and a gplots library [94]. The “color key and histogram” was used to signalize the frequency of the results obtained on the heat map matrix.

The determined activities of dehydrogenases (Deh), catalase (Cat), urease (Ure), acidic phosphatase (Pac), alkaline phosphatase (Pal), arylsulfatase (Aryl) and *β*-glucosidase (Glu) enabled the computation of the soil quality index (BA), according to Formula (2) proposed by Wyszkowska et al. [74]:

BA = Deh + Cat + Ure + Pac + Pal + Aryl + Glu(2)

The results of the determinations of *Zea mays* biomass and enzymatic activity were analyzed in terms of the normality of their distribution and the homogeneity of their variance. The analysis of variance (ANOVA) with a post hoc test (Tukey) at *P* = 0.05 was conducted separately for each soil type tested. It aimed to determine the percentage contribution (coefficient η^2^) of soil pollution with petroleum-derived products, sorbents and their interactions in changes observed in the *Zea mays* biomass and activities of individual soil enzymes. In addition, the principal component analysis (PCA) was performed in order to highlight the correlations between the results obtained. Computations were carried out using Statistica 13.0 software (Palo Alto, CA, USA) [95]. The values of the η^2^ coefficient, describing the contribution of an independent variable to the modification of the values of dependent variables, were presented on a circular diagram plotted using the Circos 0.68 package [96]. 

## 3. Results

### 3.1. Effect of Diesel Oil, Petrol and Sorbents on Zea mays Biomass

**Figure 1:**
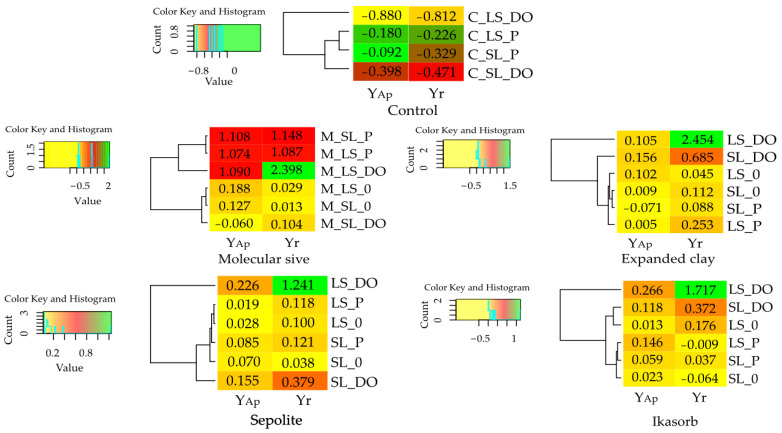
Index of the effect of DO and P and sorbents on *Zea mays* yield. LS—loamy sand; SL—sandy loam; 0—control; DO—diesel oil; P—VERVA 98 petrol; Yap—the index of influence of aerial plants; Yr—the index of influence of roots.

The analyzed petroleum-derived products significantly inhibited the growth and development of *Zea mays*. The weight of aerial part biomass produced on loamy sand (LS) polluted with DO was 88.0% lower compared to that obtained in the control, non-polluted soil, whereas the biomass produced by roots in this soil was 81.2% lower. In the case of sandy loam (SL), the respective biomass weights were 39.8% and 47.1% lower compared to those produced on the control soil (Table 2, Figure 1). Petrol was found to exert a less toxic effect on the test plants than DO. The biomass of the aerial parts produced in LS soil polluted with P was lower by 18.0% and that of roots was decreased by 22.6% compared to the non-polluted LS, whereas the respective weights of biomass produced on SL were 9.2% and 32.9% lower compared to those produced on the control soil. The ratio of biomass of the aerial parts to the biomass of roots of *Zea mays* grown on LS averaged 4.566, and the respective ratio determined for the test plants grown on SL averaged 6.247, with the biomass of the aerial parts being higher, along with an increasing root biomass. 

However, this dependency was distorted once the non-polluted soils were amended with sorbents (Table 3). The molecular sieve (M) increased the biomass of the aerial parts of *Zea mays* grown on LS by 18.8% and that of maize grown on SL by 12.7% (Table 3, Figure 1). Expanded clay (E) also exerted a positive effect on the biomass of the aerial parts on LS and that of roots on SL. The growth and development of roots were stimulated by expanded clay (E) and Ikasorb (I) but only on LS. 

The analyzed sorbents were observed to mitigate the toxic effects of DO on *Zea mays* (Table 4, Figure 1). Under their influence, the biomass of aerial parts increased by 42.2% in LS and by 9.2% in SL, on average. In LS, M had the most favorable effect on the biomass of aerial parts, while in SL E and S had the most positive effect on this parameter.All sorbents tested turned out to promote root growth and development; however, their stronger effects on root biomass were noted in LS as opposed to SL.

The effects of sorbents on the biomass of *Zea mays* grown on the soils polluted with P were significantly weaker compared to the DO-polluted soils (Table 5, Figure 1), which was due to a less toxic effect P than DO on this crop.

### 3.2. Effect of Diesel Oil, Petrol and Sorbents on the Activity of Soil Enzymes

**Figure 2:**
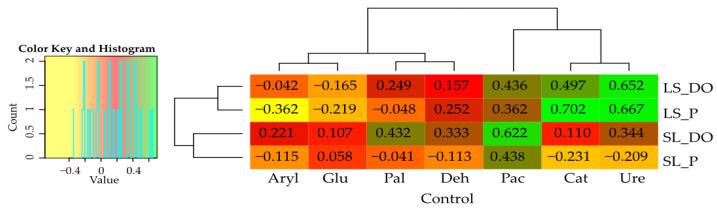

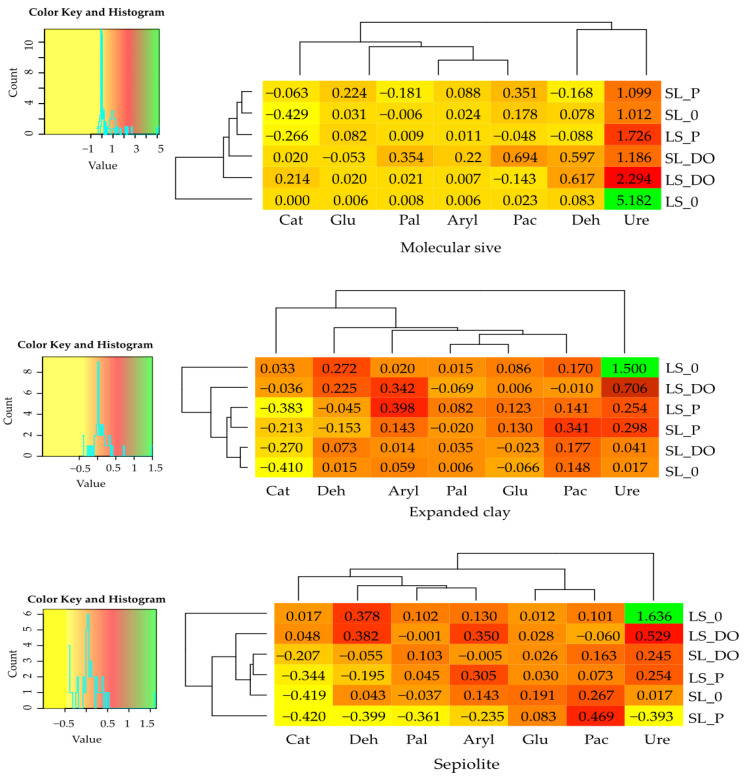
Index of the influence of DO and P and sorbents on the activity of enzymes in the soil. LS—loamy sand; SL—sandy loam; 0—control; DO—diesel oil; P—VERVA 98 petrol; Deh—dehydrogenases; Cat—catalase; Ure—urease; Pac—acid phosphatase; Pal—alkaline phosphatase; Glu—*β*-glucosidase; Aryl—arylsulfatase.

Soil pollution with DO and P significantly suppressed the activities of soil enzymes (Table 6, Figure 2). Changes observed in the enzymatic activity in both soil types were mainly due to the effect of P, which suppressed the activities of Deh, Cat, Ure, Pal and Aryl in sandy loam (SL) as well as stimulating the activities of the first three mentioned enzymes and Pac and inhibiting those of Pal, Glu and Aryl in loamy sand (LS). Soil pollution with DO significantly stimulated the activities of all analyzed enzymes in SL and almost all in LS, where it inhibited the activities of Glu and Aryl. 

The effects of the tested sorbents on enzymatic activity varied depending on soil type, sorbent and enzyme (Table 7, Figure 2). The molecular sieve turned out to be the greatest promoter of Ure activity, regardless of soil type. It suppressed Cat activity by 42.9% in SL but did not affect it in LS. In turn, it exerted a weak effect on Pal, Glu and Aryl in both soil types. Deh also proved minimally susceptible to its effects in both types of soil, as their activities increased by merely 8.3% in LS and 7.8% in SL. The other sorbents tested stimulated Deh activities but only in the lighter soil, i.e., in LS, by 27.2% (expanded clay), 37.8% (sepiolite) and 37.2% (Ikasorb). All sorbents contributed to Ure activity enhancement in LS, while causing no significant changes in its activity in SL. In turn, they increased Pac activity in the range of 10.1% to 17.0% in LS and of 14.8% to 37.8% in SL. In contrast, Pal activity was stimulated only by sepiolite and Ikasorb and only in LS. 

Enzymatic activity in the DO-polluted soils was modified by the tested sorbents to a various extent (Table 8, Figure 2). In loamy sand (SL), the activities of dehydrogenases and urease were enhanced by all sorbents tested, with the strongest stimulating effect exerted by the molecular sieve which increased Deh activity by 61.7% and Ure activity by 229.4%. This extent of activity boosting was indicated by the values of the index of influence presented in Figure 2. In LS, the molecular sieve additionally had a positive effect on catalase activity. The activity of this enzyme was also enhanced by Ikasorb. The activity of the other enzymes analyzed in LS was not significantly changed. Slightly different observations were made regarding the effects of the tested sorbents on enzymatic activity in sandy loam (SL) polluted with DO. The stimulation of Deh and Ure activities was substantially weaker. The molecular sieve stimulated Deh activities by only 19.8% and Ure activity by 62.7%. All sorbents inhibited catalase activity, whereas their usability in modifying the activities of the other enzymes was negligible.

The effects of sorbents in the soil polluted with P varied depending on the analyzed enzyme and soil type (Table 9, Figure 2).

All sorbents significantly suppressed the activities of Deh and Cat, and stimulated those of Ure and Glu in both LS and SL. In LS, all sorbents additionally stimulated activities of Pal and Aryl, whereas in SL, the added sorbents induced an increase in Pac activity. The above observations are confirmed by the values of the indices of influence presented in Figure 2. In LS, Ikasorb caused the greatest changes in Deh activities (IF_Ad_ = −0.240); expanded clay caused the greatest changes in the activities of Cat (IF_Ad_ = −0.383), Pac (IF_Ad_ = 0.141), Pal (IF_Ad_ = 0.123) and Aryl (IF_Ad_ = 0.398); and the molecular sieve caused the greatest changes in Ure activity (IF_Ad_ = 1.726). In turn, in SL, the molecular sieve had a particularly negative effect on the activity of Deh (IF_Ad_ = −0.168) and positively on the activity of Ure (IF_Ad_ = 1.099); expanded clay caused the greatest changes in the activities of Cat (IF_Ad_ = −0.213) and Aryl (IF_Ad_ = 0.143); sepiolite caused the greatest changes in Pac activity (IF_Ad_ = 0.657); and finally, the molecular sieve and Ikasorb contributed to a significant increase in Glu activity (IF_Ad_ = 0.224–0.225). Regardless of enzyme type, the molecular sieve was proven to be the most effective remediating agent in the soils polluted with DO and P. 

**Figure 3:**
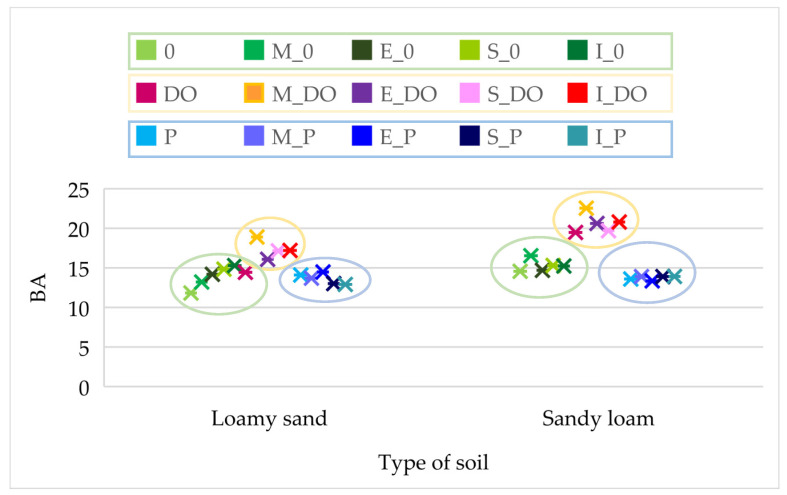
Biochemical soil quality index (BA). 0—control; M—molecular sieve; E—expanded clay; S—sepiolite; I—Ikasorb; DO—diesel oil; P—VERVA 98 petrol.

The mean value of the soil quality index (BA) reached 14.752 for loamy sand (LM) and 16.541 for sandy loam (SL), regardless of the independent variables examined (Figure 3). Soil pollution with DO caused a 22.2% increase in its value in LS and a 33.8% increase in SL, whereas soil pollution with P led to BA value increase by 19.3% in LS and its decrease by 6.6% in SL. Soil amendment with sorbents increased the values of BA index in the non-polluted soils by 9.4% (expanded clay) to 15.7% (Ikasorb) on average. In the DO-polluted soils, the observed increase ranged from 8.1% (expanded clay) to 22.1% (molecular sieve), whereas in those polluted with P, the BA values remained unaffected by the sorbents. 

**Figure 4:**
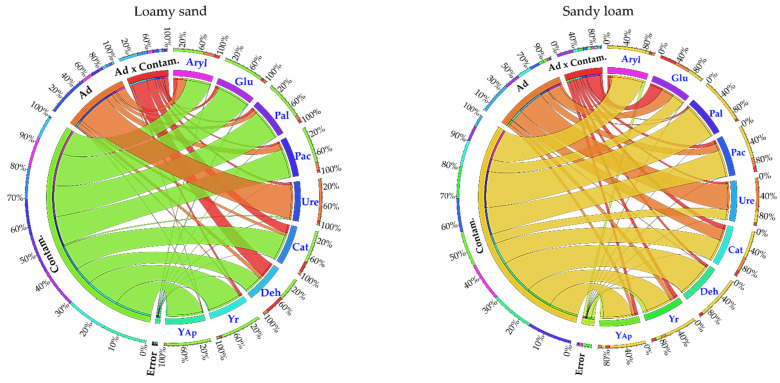
The share of independent variables (η^2^) in shaping the dependent variables in loamy sand (LS) and sandy loam (SL). Deh—dehydrogenases; Cat—catalase; Ure—urease; Pac—acid phosphatase; Pal—alkaline phosphatase; Glu—*β*-glucosidase; Aryl—arylsulfatase; Yap—the index of influence of aerial plants; Yr—the index of influence of roots; Ad—sorbents.

The sorbents tested exerted a stronger effect on the values of dependent variables in SL than in LS (Figure 4). In SL, they affected 67.8% of Ure activity, 32.3% of Glu activity, 31.1% of Cat activity and 19.5% of Pac activity, as well as 7.9% to 11.1% of *Zea mays* biomass. In LS, the sorbents contributed to barely 1.2–2.8% of changes in *Zea mays* biomass and modified 89.0% of Ure activity and 23.6% of Aryl activity, as well as over 10.0% of Deh, Pal and Pac activities.

In both soil types, pollution with DO and P had a significantly stronger effect on changes in the values of dependent variables compared to sorbents. Their contribution in the changes observed in SL ranged from 26.2% (Ure) to 94.3% (Pal) and in LS from 49.1% (Deh) to 95.7% (biomass of aerial parts of *Zea mays*). Urease activity in LS was an exception, as merely 3.2% of its value was affected by soil pollution with DO and P.

**Figure 5:**
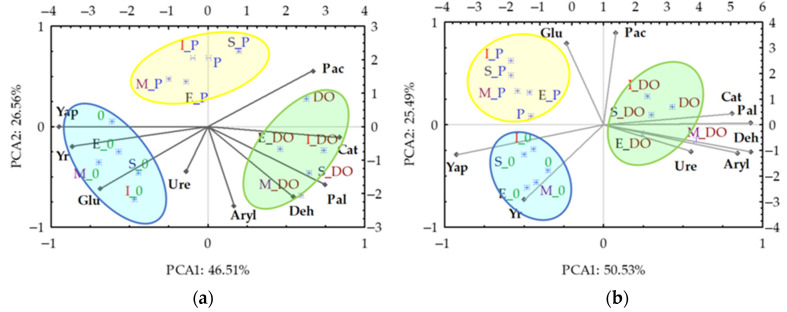
Principal component analysis loamy sand (LS) (**a**) and sandy loam (SL) (**b**). C—control; M—molecular sieve; E—expanded clay; S—sepiolite; I—Ikasorb; Deh—dehydrogenases; Cat—catalase; Ure—urease; Pac—acid phosphatase; Pal—alkaline phosphatase; Glu—*β*-glucosidase; Aryl—arylsulfatase.

Results of the principal component analysis (Figure 5) indicate that the weight of *Zea mays* biomass was negatively correlated with activities of most of the enzymes tested in both LS and SL. This negative correlation was probably due to the adverse impact of DO and P on maize growth and development and their stimulating effect on activity of enzymes such as Deh, Pal, Cat, Aryl and sometimes Ure. The adverse effect of DO on the test plant was stronger than that of P. In both soil types, in the experimental series without DO and P, the sorbents not only did not impair the growth and development of *Zea mays* but even stimulated them. Figure 5 shows three distinct groups of effects of independent variables on changes in the values of dependent variables in both soil types. The first group includes the sorbents applied on the soils non-polluted with DO and P, the second includes those used on P-polluted soils and the third one includes those used on soils polluted with DO.

## 4. Discussion

### 4.1. Response of Zea mays to Soil Contamination with Petroleum-Derived Products 

Soil pollution with petroleum-derived products poses a common problem, which requires deploying an environmentally friendly and economically effective solution [97,98]. A viable option for the decontamination of areas polluted with petroleum-derived hydrocarbons is offered by phytoremediation, especially as the biomass of plants applicable for in situ remediation can later be used to produce bioenergy [90,97]. Hence, the success of the effective remediation of soils polluted with these compounds depends on the appropriate choice of plants, considering their lasting and stable effects in the natural environment [99,100,101,102]. The choice of the plants should also be driven by the concentration and type of petroleum-derived hydrocarbons polluting the soil [98,103] and—consequently—by an appropriate phytoremediation strategy [99,104]. Native plants are preferred in this case, as they easily adapt to the local climate conditions, thereby increasing the chances for successive phytoremediation [105]. The plants intended for phytoremediation should have a well-developed root system [18,27], e.g., those from the *Poaceae* family [21,27,30,106,107,108] used for the phytoremediation of soils polluted with petroleum-derived hydrocarbons. This plant family also includes *Zea mays*, used in the present study. 

Hydrocarbons of petroleum products modify texture, structure and pore size, as well as the water–air and physicochemical properties of soil [8,28], thereby exerting a phytotoxic effect on seed germination and plant growth and development [27,28,109]. The negative impact of soil pollution from diesel oil and petrol on the growth and development of *Zea mays*, observed in the present study, could have been due to the inhibition of water and mineral salts uptake, contributing to the arrestment of metabolic processes in plants [8]. Their immediate effect on the test plants cannot be excluded either. Petroleum-derived hydrocarbons contribute to, most of all, the deformation and reduced length of roots, decreased leaf surface area, the deformation of leaf pigments, and the accumulation of H_2_O_2_, often leading to their chlorosis and necrosis [28,110,111]. The leaf chlorosis and deformation of aerial parts were also observed in the case of *Zea mays* in the present study, with diesel oil inducing a stronger effect in this respect than petrol. Our previous research also demonstrated a stronger effect of DO on *Elymus elongatus* [23] and *Lolium* [21] compared to P. According to Wyszkowska et al. [21], *Lolium hybridum* and *Phleum* proved effective in degrading hydrocarbons in the soil contaminated with diesel oil and petrol, respectively.The phytotoxicity of petroleum-derived hydrocarbons depends on petroleum product type [21,98], plant species [8,28,112,113] and the plant’s capability of the immediate absorption and metabolization of these hydrocarbons [109]. Plants absorb and then metabolize (phytotransformation, phytodegradation) organic pollutants, which are degraded by enzymes such as dehalogenase and oxygenase, secreted by plants. Plants are deemed to be the “green liver” of the biosphere [99]. Meištininkas et al. [98] have demonstrated the usability of the following legumes: *Medicago sativa*, *Lotus corniculatus* and *Melilotus albus*, in the remediation of soils polluted with heavy fuel oils, whereas Panchenko et al. [104] have recommended a mixture of *Medicago sativa* L. and *Lolium perenne* L. for the remediation of soils polluted with oils.

According to Malik et al. [114], only few plant species, e.g., *Cucurbita*, are capable of absorbing permanent pollutants (POPs) from soil. Therefore, breeding transgenic, hyperaccumulative plants encoding for POP-degrading enzymes may foster certain potential in the phytoremediation of these pollutants. The adverse impact of DO and P on the biomass of aerial parts of *Zea mays* in the present study was weaker in sandy loam (SL) than in loamy sand (LS), probably due to the stronger binding of petroleum-derived hydrocarbons with soil particles in SL than in LS and, consequently, due to their lower bioavailability [107]. The analyzed sorbents were more effective in mitigating the adverse effects of DO than P on *Zea mays*, which was due to the less toxic effects of P than DO on this crop. The positive impact of sorbents on *Zea mays* yield in soils polluted with petroleum-derived products stems from the improvement of the physical properties of soil, resulting in upgraded water–air conditions that could promote the degradation of hydrocarbons. High-porosity materials can improve the effectiveness of their bioremediation as well [115].

### 4.2. Response of Soil Enzymes to Soil Pollution with Petroleum-Derived Products 

Soil pollution with petroleum-derived products causes disorders in soil metabolism [116], which need to be considered from the perspective of sustainable development [100], health of soil and plants [29,33,34,96] and the feasibility of homeostasis restoration in the ecosystem [117,118]. For these reasons, it is essential to determine the activity of soil enzymes in real time when establishing soil quality [19,75,76,102,119,120]. In the present study, enzymatic activity was significantly modified by petroleum-derived products; however, DO stimulated it significantly in both soil types, whereas P only in LS. DO served as an energy substrate to microorganisms, owing to which we were able to observe enhanced activities of Deh, Cat, Ure, Pac and Pal in LS and SL as well as Glu and Aryl in SL. The high enzymatic activity determined in the soil polluted with this petroleum-derived product could be due to the sorption of exoenzymes in mineral and organic colloids [120,121]. In turn, the inhibiting effect of P on most analyzed enzymes could stem from modifications induced in the molecular structure of enzymes by inhibitors [122]. Petroleum-derived hydrocarbons are considered to be potent enough to disrupt the structure of protein molecules [121]. 

The modified activities of all soil enzymes were reflected in the values of the index of DO and P influence on these enzymes. The presented IF values provide an unbiased assessment of soil ecosystem’s stability [123]. The present study results demonstrate that the extent of enzymatic activity changes under the influence of DO and P depended on the fraction composition of the soil. Considering the extent of changes triggered by DO in LS, the analyzed enzymes can be ordered as follows (from the most to the least sensitive), Ure > Cat > Pac > Pal > Glu > Deh > Aryl, whereas in the case of soil pollution with P, the order is Cat > Ure > Pac = Aryl > Deh > Glu > Pal. In SL, terms of their sensitivity to soil pollution with petroleum products, the enzymes can be ordered as Pac > Pal > Ure > Deh > Aryl > Cat > Glu in the case of DO and Pac > Cat > Ure > Aryl > Deh > Glu > Pal in the case of P. The above changes in the biochemical activity of soil reflected its response to the biotic stress induced by DO and P, both being a mixture of highly hydrophobic compounds, which causes their binding with soil particles and consequently leads to their diminished bioavailability in the soil [75,124]. 

Other authors also demonstrated the destructive effect of petroleum-derived products on the biochemical properties of soil [75,125,126]. Taking into account the resistance (RS) of enzymes to diesel oil, Wyszkowska et al. [23] ordered them as follows (in descending order from the most to the least resistant): Glu > Pac > Aryl > Pal > Ure > Cat > Deh, whereas considering their resistance to unleaded petrol, their order was: Pac > Glu > Cat > Aryl > Deh > Pal > Ure. Among the analyzed enzymes, worthy of notice is *β*-glucosidase, which turned out to be one of the most resistant to the tested pollutants [91,126]. A similar observation was made in the present study, in both LS and SL. 

The exchange of matter and energy between soil, rhizospheric microorganisms and plants represents a strong and specific relationship, which allows the biochemical activity of soil to provide information highly valuable to soil health [19,75,119]. According to Qu et al. [72], dehydrogenases and polyphenolic oxidase, which provide information on the bioactivity and population of micro-organisms in the soil and play an important role in degradation of resistant organic compounds. Dehydrogenases are involved in the oxidation of organic matter [69,76], whereas polyphenolic oxidase is involved in the conversion of aromatic organic compounds into humus in the soil [83,116]. The determination of the biochemical activity may provide information about the function and structure of microbial communities in soils polluted with petroleum [83,120,123].

To achieve the present study goal, soil quality was assessed using the biochemical soil quality index (BA), which is based on activities of Deh, Cat, Ure, Pac, Pal, Glu and Aryl. The importance of these enzymes as sensitive indicators of changes in soil condition has been proven in the cases of soils polluted with heavy metals [46,74]. Computed values demonstrated that soil pollution through DO caused greater disorders in the metabolic profile of LS and SL than soil pollution through P. Presumably, DO represents a better source of nutrients to microorganisms, which in the present study could affect enzyme biosynthesis [91]. Soil polluted with petroleum may modify the structure of a bacterial community [127], whereas petroleum itself can act as a substrate for certain bacteria, facilitating soil transformation and reclamation [111,128]. 

A holistic approach should be implemented while discussing the disorders of soil metabolism. Special attention should be paid to the feasibility of restoring homeostasis in the ecosystem [128]. The present study results indicate that the biochemical activity of soil should be taken into account when planning the remediation measures undertaken to restore its productivity. Although phytoremediation is a broadly deployed and cost-effective method for the remediation of soils polluted with petroleum-derived products, it is not effective enough to significantly stimulate the biochemical activity of soils under the pressure of these xenobiotics. [129]. From an environmental perspective, it is essential to search for innovative remediation technologies useful for environments polluted with petroleum products [8,42,43] that are non-invasive and ecologically friendly [44,47]. In the present study, the phytoremediation of soils polluted with petroleum-derived products using *Zea mays* was aided by four sorbents. The molecular sieve applied to the non-polluted soil enhanced biochemical activity in both LS and SL, whereas expanded clay, sepiolite and Ikasorb enhanced it only in LS. These effects of sorbents on the activities of soil enzymes are due to their microporous structure, large specific surface areas and high ion adsorption capability [59,60,130]. It is worth emphasizing that the sorption capacity of adsorbents in relation to petroleum derivatives is usually in the range of 0.20–0.50 g·g**^−^**^1^ and the bulk density is 0.45–0.90 kg·dm**^−^**^3^ [66]. Low values of bulk density correspond to the formation of capillaries between the grains of the material, which leads to an increase in the surface available for petroleum substances. Sorption on the tested sorbents takes place by filling the available pores and capillaries between the sorbent grains. This is a capillary action that depends on the effective diameter of the capillary, the viscosity of the absorbed petroleum substance and the surface energy of the inner wall of the capillary, with the micropores present in the structure of minerals being available only for substances of low molecular weight [131]. The second mechanism of sorption concerns the outer surface of the sorbent, on which a uniform layer of petroleum substance or its irregular clusters is formed. Importantly, substances with a higher molecular weight are usually adsorbed preferentially over substances with a lower molecular weight, and the sorption capacity of sorbents is higher than petroleum substances with a higher density and viscosity [132,133]. In addition, the positive impact of sorbents on the enzymatic activity of soil reflects their effect on its physicochemical and chemical properties, including their potential to reduce hydrolytic acidity and increase the exchange capacity of the soil [134]. In the present study, all sorbents stimulated the enzymatic activity in LS polluted with DO, whereas in SL, this effect was triggered by the molecular sieve, Ikasorb and expanded clay. Sorbents had no significant effect on the enzymatic activity of the soils polluted with P, which could be due to the chemical composition of both the petroleum-derived products and sorbents themselves [128,135].

## 5. Conclusions

DO and P turned out to be toxic to *Zea mays*, with the DO found to more strongly impair its growth and development than P, and especially when maize was grown in LS rather than in SL. The sorbents proved useful in alleviating the effects of these petroleum-derived products on the growth and development of this crop, especially in LS. They were more effective in neutralizing the toxic effect of DO than P, with the molecular sieve the most effective in LS, whereas the expanded clay and sepiolite were the most effective in SL. Both soil types polluted with DO exhibited higher enzymatic activity compared to the non-polluted soils. However, similar effects of P and DO were observed only in LS. In sandy clay (SL), P was found to be a significant inhibitor of Deh, Cat, Ure, Pal and Aryl activities, while DO stimulated the activity of all enzymes in this soil. Moreover, in loamy sand (SL) contaminated with DO, all the tested sorbents increased the activity of urease to the greatest extent, with the most stimulating effect attributed to the molecular sieve. The molecular sieve stimulated the biochemical activity of both non-polluted soils. The effects of the sorbents in the soils polluted with P were negligible. Regardless of enzyme type, the molecular sieve was proven to be the most effective remediating agent in the soils polluted with DO and P, although all sorbents tested can be recommended for the remediation of soils polluted with DO, especially soils of a lower agronomic value. 

## Figures and Tables

**Table 1:** Characteristics of materials and design of the greenhouse experiment with *Zea mays*.

Parameter	Description
Loamy sand (LS)	Sandy loam: sand 0.05–2.0 mm—74.30%; silt 0.02–0.05 mm—23.69%; and clay < 0.002 mm—2.01%. 0.89 g N_tot_ kg^−1^ d.m.; 11.20 g C_org_ kg^−1^ d.m.; 164.05 mg P kg^−1^ d.m.; 53.95 mg K kg^−1^ d.m.; 46.00 mg Mg kg^−1^ d.m.; pH_KCl_—6.98; EBC—84.20 mmol (+) kg^−1^ d.m.; HAC—8.00 mmol (+) kg^−1^ d.m.; CEC—92.20 mmol (+) kg^−1^ d.m.; ACS—91.32%.
Sandy loam (SL)	Sandy loam: sand 0.05–2.0 mm—70.38%; silt 0.02–0.05 mm—27.19%; and clay < 0.002 mm—2.43%. 1.01 g N_tot_ kg^−1^ d.m.; 11.50 g C_org_ kg^−1^ d.m.; 172.73 mg P kg^−1^ d.m.; 78.85 mg K kg^−1^ d.m.; 38.00 mg Mg kg^−1^ d.m.; pH_KCl_—7.13; EBC—181.80 mmol (+) kg^−1^ d.m.; HAC—5.70 mmol (+) kg^−1^ d.m.; CEC—187.50 mmol (+) kg^−1^ d.m.; ACS—96.96%.
Molecular sieve (M)	Hydrated aluminosilicate with a micropore size of 0.3 nm. Silosiv A3 manufactured by Grace, Columbia, SC, USA, was used in the study. It is a zeolite with a three-dimensional pore system with pH_KCl_ = 8.5.
Expanded clay (E)	Lightweight ceramic aggregate produced by firing loamy clay at a temperature of about 1200 °C. It is characterized by high porosity. The aggregate used in the study was obtained from Garden Guru (Piła, Poland). It consists of 85% of particles with a size of 75 to 710 μm, with a pH_KCl_ of 7.1.
Sepiolite (S)	Naturally occurring loamy material (Mg_4_[Si_6_O_15_(OH)_2_]·6H_2_O) with a pore diameter of 1.4 nm. The study was conducted using a Sepiolite 60/100, made by the Sepiolsa Minersa Group (Guadalajara, Spain).
Ikasorb (I)	Granulate with granule diameters of 0.3–1 mm. Porous material characterized by high sorption capacity were derived from roasted diatomaceous earth. The study was conducted with Ikasorb 1850 sorbent purchased from Ikaros (Espoo, Finland)
Diesel oil VERVA (DO)	Fuel for the latest generation compression ignition engines. This product meets the stringent European requirements for the so-called “sulfur-free” fuels. Diesel oil density is 820–845 kg m^−3^. It contains a mixture of C_9_–C_25_ petroleum-derived hydrocarbons. Diesel oil was purchased at a PKN Orlen (Olsztyn, Poland) gas station. The detailed characteristics of VERVA diesel oil is provided on the following website: http://www.orlen.pl/ (accessed on 5 March 2023)
petrol VERVA 98 (P)	Unleaded motor petrol with octane number 98. Its density is 720–775 kg m^−3^. It was purchased at a PKN Orlen (Poland) gas station. Its detailed characteristics are available on the following website: http://www.orlen.pl/ (accessed on 5 March 2023)
**The Design of the Greenhouse Experiment with *Zea mays***	
Experiment Steps/Factors	Treatments
1 factor soil type	The experiment was carried out in 3.0 dm^3^ polyethylene pots with 2.5 kg portions of loamy sand (LS) or sandy loam (SL). Before the physicochemical analyses, the soil was air-dried and sieved through a screen with a 5 mm mesh diameter.
2 factor Soil contamination with of diesel oil (DO) or petrol (P)	Selected soil samples were thoroughly mixed with DO or P in doses of 0 and 7 cm^3^ of petroleum substances kg^−1^ d.m. of soil and with mineral fertilizers meeting nutritional demands of *Zea mays* and were placed in each pot.
Mineral fertilization	Nitrogen (112 mg N kg^−1^ d.m. of soil) was applied in the form of N_2_H_4_CO, phosphorus (39 mg P kg^−1^ d.m. of soil) in the form of KH_2_PO_4_ and potassium (112 mg K kg^−1^ d.m. of soil); likewise, the dose of phosphorus, in the form of KH_2_PO_4_ up to 112 mg kg^−1^ d.m. of soil was completed with KCl. *Zea mays* was also fertilized with a magnesium dose of 15 mg kg^−1^ d.m. of soil, in the form of MgSO_4_ × 7H_2_O.
3 factor sorbent type	All sorbents, namely control, molecular sieve, expanded clay, sepiolite and Ikasorb, were used in amounts of 10 g·kg^−1^ d.m. of soil.
Maximum water capacity	Subsequently, 7 cm^3^ of a suitable petroleum substance, measured with a cylinder, was added to the individual pots and mixed with the soil material in order to homogenize the samples. Once the soil had been packed in pots, its moisture content was brought to 60% of the maximum water capacity.
An experimental plant	After 7 days, the soil was sown with eight seeds of *Zea mays* PR39H32 (variety registered in the European Union). Five plants were left in each pot after emergence. Vegetation growth was continued for 60 days, while maintaining the stable moisture content of the soil using distilled water. The pots were located in random complete blocks on tables.

EBC—sum of exchangeable base cations; HAC—hydrolytic acidity; CEC—cation exchange capacity; ACS—alkaline cation saturation.

**Table 2:** The effect of soil contamination with diesel oil (DO) and petrol (P) on *Zea mays* biomass.

Objects	Loamy Sand (LS)			Sandy Loam (SL)		
	Aerial Parts (Ap)	Roots (R)	Ap/R	Aerial Parts (Ap)	Roots (R)	Ap/R
	g d.m. of pot^−1^			g d.m. of pot^−1^		
C	40.712 ^a^	8.818 ^a^	4.617	46.708 ^a^	8.533 ^a^	5.474
DO	4.903 ^c^	1.656 ^c^	2.961	28.122 ^c^	4.513 ^c^	6.231
P	33.378 ^b^	6.826 ^b^	4.890	42.422 ^b^	5.723 ^b^	7.413
Average	26.331	5.767	4.566	39.084	6.256	6.247

C—control; DO—diesel oil; P—VERVA 98 petrol. Homogeneous groups denoted with letters (a–c) were calculated separately for each part of the plant and each kind of soil.

**Table 3:** The influence of applied sorbents on *Zea mays* biomass in soils uncontaminated with petroleum products.

Object	Loamy Sand (LS)			Sandy Loam (SL)		
	Aerial Parts (Ap)	Roots (R)	Ad/R	Aerial Parts (Ap)	Roots (R)	Ad/R
	g d.m. of pot^−1^			g d.m. of pot^−1^		
C	40.712 ^c^	8.818 ^b^	4.617	46.708 ^c^	8.533 ^ab^	5.474
M	48.363 ^a^	9.074 ^b^	5.330	52.658 ^a^	8.646 ^ab^	6.090
E	44.856 ^b^	9.211 ^ab^	4.870	47.138 ^c^	9.489 ^a^	4.968
S	41.859 ^c^	9.699 ^ab^	4.316	49.990 ^b^	8.854 ^ab^	5.646
I	41.255 ^c^	10.369 ^a^	3.979	47.779 ^c^	7.986 ^b^	5.983
Average	43.409	9.434	4.622	48.855	8.702	5.614

C—control; M—molecular sieve; E—expanded clay; S—sepiolite; I—Ikasorb. Homogeneous groups denoted with letters (a–c) were calculated separately for each part of the plant and each kind of soil.

**Table 4:** Effect of sorbents on the amount of *Zea mays* biomass grown on soils contaminated with diesel oil (DO).

Object	Loamy Sand (LS)			Sandy Loam (SL)		
	Aerial Parts (Ap)	Roots (R)	Ad/R	Aerial Parts (Ap)	Roots (R)	Ad/R
	g d.m. of pot^−1^			g d.m. of pot^−1^		
C	4.903 ^c^	1.656 ^c^	2.961	28.122 ^bc^	4.513 ^c^	6.231
M	10.249 ^a^	5.629 ^a^	1.821	26.434 ^c^	4.982 ^c^	5.306
E	5.417 ^bc^	5.720 ^a^	0.947	32.500 ^a^	7.603 ^a^	4.275
S	6.012 ^b^	3.712 ^b^	1.620	32.494 ^a^	6.223 ^b^	5.222
I	6.205 ^b^	4.501 ^b^	1.379	31.449 ^ab^	6.192 ^b^	5.079
Average	6.557	4.244	1.745	30.200	5.903	5.222

C—control; M—molecular sieve; E—expanded clay; S—sepiolite; I—Ikasorb. Homogeneous groups denoted with letters (a–c) were calculated separately for each part of the plant and each kind of soil.

**Table 5:** Effect of sorbents on the amount of *Zea mays* biomass grown on soils contaminated with petroleum (P).

Objects	Loamy Sand (LS)			Sandy Loam (SL)		
	Aerial Parts (Ap)	Roots (R)	Ad/R	Aerial Parts (Ap)	Roots (R)	Ad/R
	g d.m. of pot^−1^			g d.m. of pot^−1^		
C	33.378 ^b^	6.826 ^b^	4.890	42.422 ^bc^	5.723 ^c^	7.413
M	35.863 ^ab^	7.424 ^b^	4.831	47.003 ^a^	6.572 ^a^	7.152
E	33.542 ^b^	8.555 ^a^	3.921	39.426 ^c^	6.226 ^abc^	6.332
S	34.018 ^b^	7.634 ^ab^	4.456	46.042 ^a^	6.415 ^ab^	7.177
I	38.242 ^a^	6.767 ^b^	5.651	44.933 ^ab^	5.934 ^bc^	7.572
Average	35.009	7.441	4.750	43.965	6.174	7.129

C—control; M—molecular sieve; E—expanded clay; S—sepiolite; I—Ikasorb. Homogeneous groups denoted with letters (a–c) were calculated separately for each part of the plant and each kind of soil.

**Table 6:** Influence of soil contamination with diesel oil (DO) and petrol (P) on the activity of enzymes in 1 kg of d.m. soil h**^−^**^1^.

Kind of Soil	Object	Deh μM TFF	Cat M O_2_	Ure mM N-NH_4_	Pac	Pal	Glu	Aryl mM PNS
					**mM PNP**			
Loamy sand (LS)	0	5.901 ^c^	0.227 ^c^	0.160 ^b^	2.473 ^c^	2.086 ^b^	0.571 ^a^	0.401 ^a^
	DO	6.824 ^b^	0.339 ^b^	0.264 ^b^	3.551 ^a^	2.606 ^a^	0.476 ^b^	0.385 ^b^
	P	7.388 ^a^	0.386 ^a^	0.266 ^a^	3.369 ^b^	1.986 ^c^	0.446 ^c^	0.256 ^c^
	Average	6.705	0.317	0.230	3.131	2.226	0.498	0.347
Sandy loam (SL)	0	8.550 ^b^	0.393 ^b^	1.238 ^b^	1.011 ^c^	1.619 ^b^	0.986 ^b^	0.765 ^b^
	DO	11.395 ^a^	0.437 ^a^	1.664 ^a^	1.640 ^a^	2.318 ^a^	1.092 ^a^	0.934 ^a^
	P	7.588 ^c^	0.303 ^c^	0.979 ^c^	1.454 ^b^	1.553 ^b^	1.044 ^ab^	0.677 ^c^
	Average	9.178	0.378	1.294	1.368	1.830	1.040	0.792

0—control; DO—diesel oil; P—VERVA 98 petrol; Deh—dehydrogenases; Cat—catalase; Ure—urease; Pac—acid phosphatase; Pal—alkaline phosphatase; Glu—*β*-glucosidase; Aryl—arylsulfatase. Homogeneous groups denoted with letters (a–c) were calculated separately for each kind of enzyme and each kind of soil.

**Table 7:** Influence of the applied sorbents on the activity of enzymes in soils uncontaminated with petroleum products in 1 kg of d.m. soil h**^−^**^1^.

Type of Soil	Object	Deh μM TFF	Cat M O_2_	Ure mM N-NH_4_	Pac	Pal	Glu	Aryl mM PNS
					mM PNP			
Loamy sand (LS)	C	5.901 ^b^	0.227 ^b^	0.160 ^d^	2.473 ^e^	2.086 ^c^	0.571 ^b^	0.401 ^b^
	M	6.392 ^b^	0.227 ^b^	0.988 ^a^	2.530 ^d^	2.104 ^c^	0.574 ^b^	0.404 ^b^
	E	7.509 ^a^	0.234 ^ab^	0.399 ^c^	2.893 ^a^	2.117 ^c^	0.620 ^a^	0.409 ^b^
	S	8.130 ^a^	0.231 ^b^	0.421 ^c^	2.724 ^c^	2.299 ^b^	0.577 ^b^	0.453 ^a^
	I	8.096 ^b^	0.246 ^a^	0.595 ^b^	2.842 ^b^	2.392 ^a^	0.632 ^a^	0.477 ^a^
	Average	7.206	0.233	0.513	2.692	2.200	0.595	0.429
Sandy loam (SL)	C	8.550 ^b^	0.393 ^a^	1.238 ^b^	1.011 ^d^	1.619 ^a^	0.986 ^c^	0.765 ^b^
	M	9.222 ^a^	0.225 ^c^	2.491 ^a^	1.191 ^c^	1.609 ^a^	1.016 ^c^	0.783 ^b^
	E	8.678 ^b^	0.232 ^bc^	1.260 ^b^	1.160 ^c^	1.628 ^a^	0.921 ^d^	0.810 ^b^
	S	8.919 ^ab^	0.229 ^c^	1.260 ^b^	1.281 ^b^	1.559 ^b^	1.174 ^a^	0.874 ^a^
	I	8.720 ^b^	0.244 ^b^	1.325 ^b^	1.393 ^a^	1.654 ^a^	1.087 ^b^	0.818 ^b^
	Average	8.818	0.265	1.515	1.207	1.614	1.037	0.810

C—control; M—molecular sieve; E—expanded clay; S—sepiolite; I—Ikasorb; Deh—dehydrogenases; Cat—catalase; Ure—urease; Pac—acid phosphatase; Pal—alkaline phosphatase; Glu—*β*-glucosidase; Aryl—arylsulfatase. Homogeneous groups denoted with letters (a–d) were calculated separately for each kind of enzyme and each type of soil.

**Table 8:** The effect of the applied sorbents on the activity of enzymes in soils contaminated with diesel oil (DO) in 1 kg of d.m. soil h**^−^**^1^.

Type of Soil	Object	Deh μM TFF	Cat M O_2_	Ure mM N-NH_4_	Pac	Pal	Glu	Aryl mM PNS
					mM PNP			
Loamy sand (LS)	C	6.824 ^d^	0.339 ^c^	0.264 ^c^	3.551 ^b^	2.606 ^c^	0.476 ^a^	0.385 ^c^
	M	11.033 ^a^	0.412 ^a^	0.869 ^a^	3.044 ^d^	2.662 ^b^	0.486 ^a^	0.387 ^c^
	E	8.357 ^c^	0.327 ^d^	0.450 ^b^	3.517 ^b^	2.427^d^	0.479 ^a^	0.516 ^a^
	S	9.429 ^b^	0.356 ^b^	0.404 ^b^	3.338 ^c^	2.604 ^c^	0.490 ^a^	0.519 ^a^
	I	9.144 ^b^	0.408 ^a^	0.404 ^b^	3.608 ^a^	2.713 ^a^	0.495 ^a^	0.422 ^b^
	Average	8.957	0.368	0.478	3.412	2.602	0.485	0.446
Sandy loam (SL)	C	11.395 ^bc^	0.437 ^a^	1.664 ^c^	1.640 ^c^	2.318 ^c^	1.092 ^bc^	0.934 ^a^
	M	13.653 ^a^	0.401 ^b^	2.707 ^a^	1.712 ^bc^	2.193 ^d^	0.934 ^d^	0.933 ^a^
	E	12.223 ^b^	0.319 ^d^	1.732 ^c^	1.930 ^a^	2.399 ^b^	1.067 ^c^	0.947 ^a^
	S	10.765 ^c^	0.346 ^c^	2.072 ^b^	1.906 ^a^	2.558 ^a^	1.120 ^b^	0.929 ^a^
	I	12.558 ^ab^	0.398 ^b^	1.437 ^d^	1.822 ^ab^	2.432 ^b^	1.257 ^a^	0.882 ^a^
	Average	12.119	0.380	1.922	1.802	2.380	1.094	0.925

C—control; M—molecular sieve; E—expanded clay; S—sepiolite; I—Ikasorb; Deh—dehydrogenases; Cat—catalase; Ure—urease; Pac—acid phosphatase; Pal—alkaline phosphatase; Glu—*β*-glucosidase; Aryl—arylsulfatase. Homogeneous groups denoted with letters (a–d) were calculated separately for each kind of enzyme and each kind of soil.

**Table 9:** Influence of the applied sorbents on the activity of enzymes in soils contaminated with petroleum (P) in 1 kg of d.m. soil h**^−^**^1^.

Type of Soil	Object	Deh μM TFF	Cat M O_2_	Ure mM N-NH_4_	Pac	Pal	Glu	Aryl mM PNS
					mM PNP			
Loamy sand (LS)	C	7.388 ^a^	0.386 ^a^	0.266 ^c^	3.369 ^c^	1.986 ^d^	0.446 ^c^	0.256 ^c^
	M	6.736 ^b^	0.283 ^b^	0.726 ^a^	3.208 ^d^	2.003 ^d^	0.483 ^ab^	0.259 ^c^
	E	7.058 ^ab^	0.238 ^d^	0.334 ^c^	3.842 ^a^	2.148 ^b^	0.501 ^a^	0.358 ^a^
	S	5.945 ^c^	0.253 ^cd^	0.334 ^c^	3.616 ^b^	2.076 ^c^	0.459 ^bc^	0.334 ^ab^
	I	5.616 ^c^	0.261 ^c^	0.421 ^b^	3.640 ^b^	2.198 ^a^	0.456 ^bc^	0.319 ^b^
	Average	6.549	0.284	0.416	3.535	2.082	0.469	0.305
Sandy loam (SL)	C	7.588 ^a^	0.303 ^a^	0.979 ^c^	1.454 ^d^	1.553 ^a^	1.044 ^c^	0.677 ^b^
	M	6.312 ^c^	0.283 ^b^	2.055 ^a^	1.964 ^c^	1.271 ^b^	1.277 ^a^	0.737 ^ab^
	E	6.426 ^c^	0.238 ^d^	1.271 ^b^	1.949 ^c^	1.522 ^a^	1.180 ^b^	0.774 ^a^
	S	6.854 ^b^	0.253 ^c^	1.009 ^c^	2.409 ^a^	1.482 ^b^	1.183 ^b^	0.714 ^ab^
	I	6.732 ^b^	0.261 ^c^	1.075 ^c^	2.328 ^b^	1.560 ^a^	1.278 ^a^	0.675 ^b^
	Average	6.782	0.268	1.278	2.021	1.478	1.192	0.715

C—control; M—molecular sieve; E—expanded clay; S—sepiolite; I—Ikasorb; Deh—dehydrogenases; Cat—catalase; Ure—urease; Pac—acid phosphatase; Pal—alkaline phosphatase; Glu—*β*-glucosidase; Aryl—arylsulfatase. Homogeneous groups denoted with letters (a–d) were calculated separately for each kind of enzyme and each type of soil.

## Data Availability

Data are available by contacting the authors.

## Abbreviations

0/C—control; M—molecular sieve; E—expanded clay; S—sepiolite; I—Ikasorb; Ad—sorbents; Deh—dehydrogenases; Cat—catalase; Ure—urease; Pac—acid phosphatase; Pal—alkaline phosphatase; Glu—*β*-glucosidase; Aryl—arylsulfatase; BA—biochemical soil quality index; Yap—the index of influence of aerial plants; Yr—the index of influence of roots; DO—diesel oil; P—VERVA 98 petrol; LS—loam sandy; SL—sandy loam.
